# Case Report: A Novel Homozygous Variant Identified in a Chinese Patient With Benign Recurrent Intrahepatic Cholestasis-Type 1

**DOI:** 10.3389/fmed.2021.705489

**Published:** 2021-08-18

**Authors:** Huayu Chen, Dongbo Wu, Wei Jiang, Ting Lei, Changli Lu, Taoyou Zhou

**Affiliations:** ^1^Center of Infectious Diseases, West China Hospital, Sichuan University, Chengdu, China; ^2^Department of Pathology, West China Hospital, Sichuan University, Chengdu, China

**Keywords:** intrahepatic cholestasis, BRIC, diagnosis, treatment, ATP8B1 gene, case report

## Abstract

Benign recurrent intrahepatic cholestasis (BRIC) is a rare hereditary cholestatic liver disorder. Accurate diagnosis and timely interventions are important in determining outcomes. Besides clinical and pathologic diagnosis, genetic study of BRIC remains limited. Here, we report a young man enduring recurrent jaundice and severe pruritus for 15 years. The increased level of direct bilirubin was the main biochemical abnormality, and the work-up for common causes of jaundice were unremarkable. Liver biopsy showed extensive cholestasis of hepatocytes in zone 3. The novel homozygous variant including *c.1817T* > *C* and *p.I606T* was detected on his *ATP8B1*gene. The patient was finally diagnosed with BRIC-1. His symptoms were relieved, and liver function tests returned to normal after taking ursodeoxycholic acid. This case provides a different perspective to the methodology employed when dealing with cases of jaundice and helping diagnose rare diseases.

## Introduction

Benign recurrent intrahepatic cholestasis (BRIC) is a rare autosomal recessive hereditary metabolic disorder characterized by recurrent episodes of cholestatic jaundice. The first case of BRIC was described as obstructive jaundice by Summerskill et al. in 1959 (1). BRIC is a rare genetic disorder characterized by intermittent episodes of jaundice and pruritus. The first episode may occur from age 1–59 years, and it frequently occurs in the first two decades of life (2). The exact prevalence of BRIC remains unknown, but the estimated incidence is ~1 in 50,000–100,000 people worldwide (3). Episodes of BRIC can be spontaneous or triggered by some factors such as infection, pregnancy, and stress (4, 5).

Numerous factors have been proven to induce liver injury, and their common manifestation is jaundice. Inherited metabolic jaundice is relatively rare and its clinical presentation is complex, which makes it easy to miss or to be misdiagnosed. According to the locations of genetic variants, it is classified into two subtypes including BRIC type 1 (BRIC-1) and BRIC type 2 (BRIC-2).

Owing to a variant of the *ATPase phospholipid transporting 8B1(ATP8B1)* gene, familial intrahepatic cholestasis 1 (FIC1) in BRIC-1 is deficient, and this could weaken the function of the bile salt export pump (BSEP) that is responsible for transporting bile into the canaliculi (2, 3, 6, 7). FIC1 is probably associated with the stability of the plasma membrane, and an associated disturbance might influence the function of other transmembrane proteins including BSEP. Moreover, a variant of the *ABCB11* gene in BRIC-2 can decrease the count of BSEP transporters (2, 3, 6). Both variants could result in cholestasis by impairing the function of the BSEP directly or indirectly. The gene variant in *ATP8B1* including *c.1817T*>*C* and *p.I606T* was first reported in our patient. According to the results of liver biopsies and long-term prognosis of BRIC patients, few cases progressed to liver failure. Nevertheless, there still is an enormous challenge with respect to its diagnosis and timely therapy. This case may provide a different perspective to the methodology employed when dealing with cases of jaundice and helping diagnose rare diseases.

## Case Report

A young male teacher who visited our hospital in November 2017 has complained of recurrent jaundice and severe pruritus experienced for more than 15 years. His symptoms first occurred in 2002 while he was still 19 years old. He denied experiencing fever, chills, vomiting, abdominal pain, and weight loss. He also denied history of similar episodes among his family members. Thus, he visited a local hospital. His liver functional test showed total bilirubin (TB) level of over 50.0 μmol/L and slightly elevated transaminase level. Computed tomography of the abdomen showed no remarkable findings. His TB level increased gradually, reaching a maximum of 500.0 μmol/L during hospitalization. His condition improved gradually, and liver function tests normalized after 4 months. He experienced seven similar episodes over a span of 15 years, which sometimes aggravated after administering penicillin and ibuprofen for treating common cold. Two weeks before visiting our hospital, he took “Vitamin C Yinqiao Tablets,” which is a traditional Chinese medicine for cold, for sore throat for 3 days. Further, he developed itchy skin (pruritis), his skin and sclera became increasingly yellow, and his urine and stool turned dark brown. On his visit to the local hospital, his liver functional tests revealed alanine aminotransferase (ALT) of 242.0 U/L, aspartate aminotransferase (AST) of 102.0 U/L, TB of 73.2 μmol/L, and direct bilirubin (DB) of 59.4 μmol/L. On admission, physical examination showed his skin and sclera were moderately yellow. His entire abdomen was soft and nontender. He neither had hepatomegaly nor splenomegaly. No spider angiomas or palmar erythema was observed on his skin. Dynamic changes of his liver test are shown in Table 1. Although his blood ammonia level was 104.0 μmol/L (normal range, 9.0–33.0 μmol/L), he did not show signs of central nervous system involvement or hepatic encephalopathy. His urine tested positive for bilirubin. Results of blood and fecal routine examination and coagulation functions were within normal ranges. The alpha-fetoprotein level was normal. Serum makers were negative for hepatitis A, B, C, D, and E viruses. The screening for toxoplasmosis, rubella, cytomegalovirus, and herpes simplex virus infections was negative. Deoxyribonucleic acid detection of Epstein-Barr virus and human cytomegalovirus were both negative. Autoimmune antibodies including anti-mitochondria antibody, anti-hepatorenal microsomal antibody, anti-hepatocyte solute antibody, and anti-soluble liver antigen antibody were negative. Serum levels of copper and ceruloplasmin were normal. Serum levels of thyroid hormones were normal. Ultrasonography revealed a normal liver echo texture and non-dilated biliary system. Magnetic resonance cholangiopancreatography confirmed the absence of dilatation of any intrahepatic or extrahepatic bile duct, except for mild splenomegaly.

**Table 1:** The dynamic changes of liver function.

**Date**	**TB**	**DB**	**ALT**	**AST**	**ALP**	**GGT**	**ALB**	**GLB**
**Date**	**(μmol/L)**	**(μmol/L)**	**(IU/L)**	**(IU/L)**	**(IU/L)**	**(IU/L)**	**(g/L)**	**(g/L)**
2015.08.05	530.3	448.3	56	44	377	32	39.2	24.8
2017.10.03	73.2	59.4	242	102	136	23	41.2	24.6
2017.11.03	115.6	96.1	144	56	188	12	38.9	25.7
2017.11.10	357.6	281.8	46	37	265	18	40	24.7
2017.11.15	573.8	424.7	34	39	276	21	40.3	22.9
2017.11.21	564.4	436.8	29	24	237	47	37.3	16.8
2017.11.26	529.5	425.5	20	17	315	45	35.9	17.4
2020.01.01	14.3	4.2	31	25	89	14	36.9	17.2

*TB, total bilirubin; DB, direct bilirubin; ALT, alanine aminotransferase; AST, aspartate aminotransferase; ALP, alkaline phosphatase; GGT, γ-glutamyl transpeptidase; ALB, albumin; GLB, globulin*.

**Figure 1:**
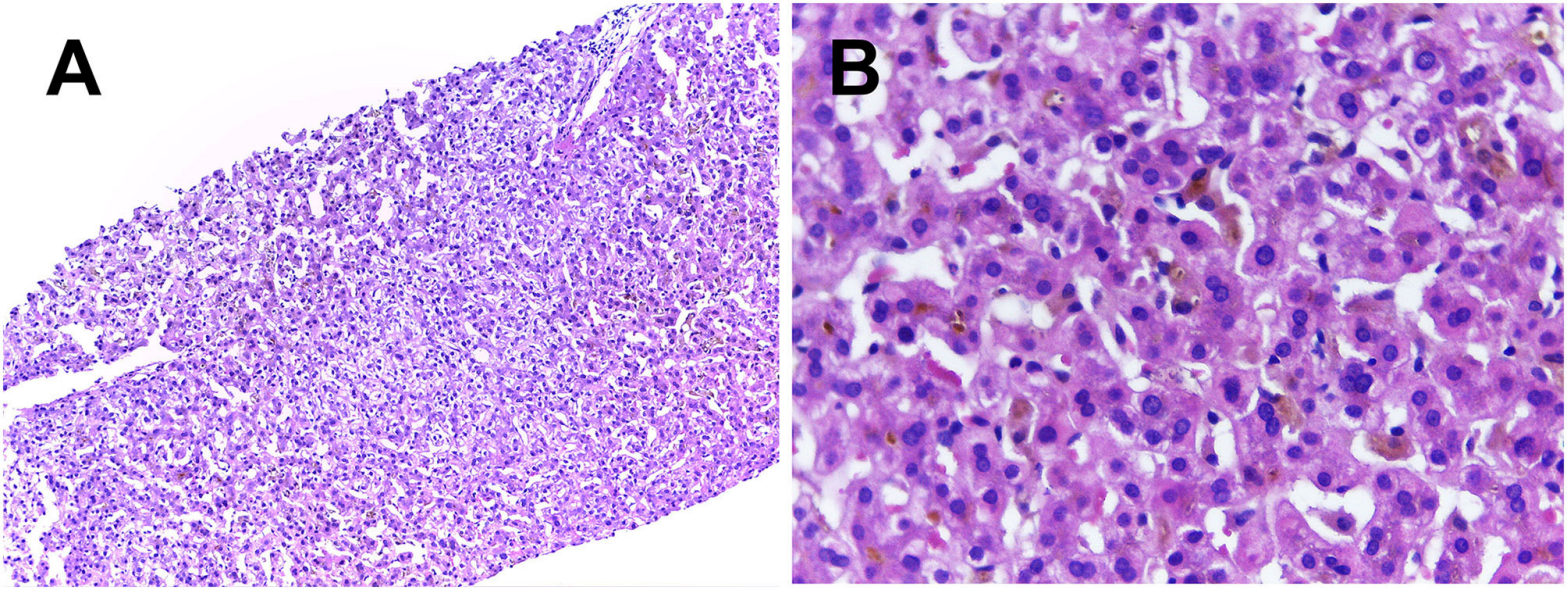
Liver histology showed normal lobular architecture with few lymphocytes, monocytes and neutrophils infiltrating the portal areas were seen in **(A)** (H&E × 100). Extensive cholestasis of hepatocytes and the formation of bile embolism were better observed at a higher magnification in the centrilobular zone 3 on **(B)** (H&E × 400).

With the collected evidence, common liver diseases causing jaundice were excluded. However, the cause of his obstructive cholestasis still remained unclear. After obtaining his consent, liver biopsy was performed. The result of biopsy reported mostly normal liver lobule structures and extensive cholestasis of hepatocytes in zone 3 accompanied by the formation of bile embolism; few lymphocytes, monocytes, and neutrophils were found infiltrating the portal areas. Positive cytokeratin 7 staining indicated slight proliferation of small bile ducts and cholestatic state in a part of the hepatocytes. Immunohistochemical staining of hepatitis B surface antigen and hepatitis B core antigen, periodic acid-Schiff staining, copper staining, and iron staining were negative (Figure 1).

**Figure 2:**
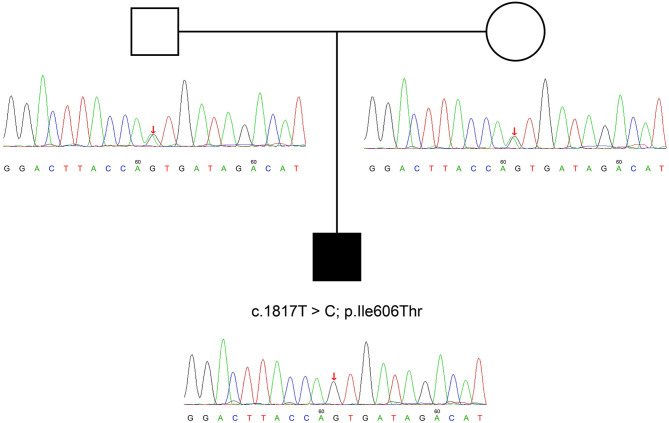
*ATP8B1* c.1817T >C variant identified in the parents of our patient. We can deduce that his variant is potentially derived from his parents.

On further molecular analysis of whole-exome sequencing for the *ATP8B1* gene, homozygous variants, including *c.1817T*>*C* and *p.I606T* were revealed. This is a novel variant site that has been searched on certain databases including Genome Aggregation Database (gnomAD), Human Gene Variant Database, and Clinvar of NCBI. A familial genetic testing by sanger sequencing showed heterozygosity in his parents' *ATP8B1* genes (Figure 2).

The diagnosis of intrahepatic cholestasis was made with total evaluation of case history, clinical features, examination, and liver biopsy as per the criteria proposed by Luketic and Shiffman (8). The patient was finally diagnosed with BRIC-1.

The patient was prescribed with ursodeoxycholic acid (UDCA). His symptoms relieved, and his liver function recovered 1 month after his discharge from the hospital. During the follow-up period of about 2 years (January 2020), the patient did not show any signs of jaundice or pruritus, and liver function tests were always normal (ALT, 31 U/L; AST, 25 U/L; TB, 14.3 μmol /L; DB, 4.2 μmol/L; gamma glutamyl transpeptidase [GGT], 14 U/L). His liver function tests in May 2021 were normal.

## Discussion

Herein, we report a young male patient with recurrent episodic jaundice and itching without obvious anatomical abnormalities on liver imaging. The common causes of liver injury such as viral infections, autoimmune diseases, alcoholic liver disease, and drug-induced liver disease were excluded, and his liver biopsy showed extensive cholestasis of hepatocytes in zone 3. Along with the result of *ATP8B1* gene variant, he was finally diagnosed with BRIC-1. With a more than 3 years follow-up of our BRIC1 patient, we found a mild course of disease associated with c.1817T>C, p.(I606T) homozygosity based on clinical and biochemical phenotype.

BRIC is divided into two sub-types, namely, BRIC-1 and BRIC-2. Patients with BRIC-1 usually have a variant in the *ATP8B1* gene (chromosome 18q21-22), which encodes a protein called FIC1. The function of FIC1 (9) protein remains unknown; however, based on various studies, it has been theorized that in *ATP8B1-*deficient mice it acts as an amino-phospholipid translocase that transports phospholipids from outside the canalicular membrane to inside (10). BRIC-2 is associated with a variant in the *ABCB11* gene (chromosome 2q24) that is in charge of encoding BSEP (11). As its name suggests, BSEP is responsible for exporting bile into the canaliculi from the hepatocytes (2). BRIC is mainly characterized by episodic cholestasis and severe pruritus accompanied by asymptomatic intervals for several months to years. Laboratory indicators are consistent with intrahepatic cholestasis with or without slightly elevated GGT level. Liver histology indicates intralobular cholestasis and bile plugs in the canaliculi. The clinical manifestations of our patient were consistent with the above description. Having ruled out the common causes of jaundice, we can make a diagnosis according to the set of criteria proposed by Luketic and Shiffman in 2004 (8). Even if the patient has had repeated seizures, a majority of BRIC patients still had normal liver structure.

Few BRIC cases have been reported to have developed into progressive familial intrahepatic cholestasis (PFIC), wherein the variants of *ATP8B1* and *ABCB11* gene were also observed (PFIC types 1 and 2, respectively) (12). PFIC is also a type of autosomal recessive disorder characterized by intrahepatic cholestasis with a strong phenotype and poor prognosis because it usually progresses into end-stage liver disease (13). In other words, manifestations of PFIC are often observed in infancy or early childhood and results in end-stage liver disease, death, or treatment by liver transplantation (14). PFIC is classified into six subtypes based on the genetic defects involved in bile transport (7, 14). However, these cholestatic diseases in which synthesis or canalicular secretion of bile salts is virtually absent should be ruled out (15). The classifications of BRIC and PFIC are presented in Table 2.

**Table 2:** Classifications of BRIC and PFIC.

**Benign recurrent intrahepatic cholestasis (BRIC)**		
Types	Mutant gene	Possible complications
BRIC1	*ATP8B1*	Pancreatitis
BRIC2	*ABCB11*	Cholelithiasis
**Progressive familial intrahepatic cholestasis (PFIC)**		
Type	Mutant gene	GGT level
*PFIC1*	*ATP8B1*	Low GGT
*PFIC2*	*ABCB11*	Low GGT
*PFIC3*	*ABCB4*	High GGT
*PFIC4*	*TJP2*	Low GGT
*PFIC5*	*NR1H4*	Low GGT
*PFIC6*	*MYO5B*	Low GGT

*GGT, γ-glutamyl transpeptidase; ATP8B1, ATPase phospholipid transporting 8B1; ABCB11, ATP binding cassette subfamily B member 11; ABCB4, ATP binding cassette subfamily B member; TJP2, recombinant junction protein 2; NR1H4, nuclear receptor subfamily1 group H member 4; MYO5B, Myosin VB*.

Similar to the majority of other genetic and metabolic diseases, no effective methods have been established to cure BRIC. The aim is to relieve symptoms, shorten episodes of discomfort, and prevent complications (6). In addition, there is no specific treatment based on different subtypes at present. A few studies have reported some various experiences of different drug administrations or surgical treatment (16). There was a case preventing successfully the development of further cholestatic episodes on a patient with severe BRIC by colestyramine treatment (17). There was a case demonstrating that a patient with BRIC-2 response to corticosteroids (18). Conceivably, through the development of the gene research of this disease, we can select effective treatment for different subtypes of BRIC.

Current therapeutic methods for BRIC patients comprise both drugs and endoscopic nasobiliary drainage (16). Drugs predominantly contain UDCA and rifampicin besides fat-soluble vitamins (2, 3, 6, 16). UDCA can stimulate hepatobiliary secretion of bile salts due to the possibility of promoting bile flow to the canalicular membrane via the activation of a complex signaling network (19, 20). In addition, anti-apoptotic effects of UDCA conjugates may contribute to protection of hepatocytes (6). Therefore, it may improve serum transaminase levels and relieve pruritus. In the present case, the episode was mainly relieved by UDCA. In addition, rifampicin is regarded to be as effective in improving pruritus and in halting a cholestatic episode in BRIC patients (21). As a temporary diversion of bile established by endoscope, the nasobiliary drain could also be used in BRIC patients with intractable pruritus during long-lasting cholestatic episodes (16).

## Conclusion

Jaundice is common but relatively difficult for making an etiological diagnosis after ruling out common causes. Therefore, genetic diseases and metabolic disorders should be considered. BRIC is a rare inherited metabolic disorder characterized by recurrent episodes of cholestatic jaundice. As a chronic and recurrent disease, early accurate diagnosis and timely therapy could obtain a relatively better prognosis. Liver biopsy and genetic detection play a significant role in the diagnosis and prognosis of genetic metabolic diseases.

## Data Availability Statement

The original contributions presented in the study are included in the article/supplementary material, further inquiries can be directed to the corresponding authors.

## Ethics Statement

Written informed consent was obtained from the individual(s) for the publication of any potentially identifiable images or data included in this article. Our patient agreed and signed the informed consent.

## Author Contributions

HC and DW: writing-original draft. CL and TZ: writing-review and revised. DW, WJ, and TL: figures-making. HC, DW, WJ, TL, CL, and TZ: data curation. All authors read and approved the final version of the manuscript.

## Conflict of Interest

The authors declare that the research was conducted in the absence of any commercial or financial relationships that could be construed as a potential conflict of interest.

## Publisher's Note

All claims expressed in this article are solely those of the authors and do not necessarily represent those of their affiliated organizations, or those of the publisher, the editors and the reviewers. Any product that may be evaluated in this article, or claim that may be made by its manufacturer, is not guaranteed or endorsed by the publisher.
